# Development and Validation of USM-Insulin Adherence Module for Patients with Type 2 Diabetes Mellitus

**DOI:** 10.21315/mjms2024.31.2.9

**Published:** 2024-04-23

**Authors:** Aida Maziha Zainudin, Aida Hanum Ghulam Rasool, Mohd Zarawi Mat Nor, Norul Badriah Hassan, Rosediani Muhamad, Wan Mohd Izani Wan Mohamed

**Affiliations:** 1 Department of Pharmacology, School of Medical Sciences, Universiti Sains Malaysia, Kelantan, Malaysia; 2 Hospital Universiti Sains Malaysia, Kelantan, Malaysia; 3 Department of Medical Education, School of Medical Sciences, Universiti Sains Malaysia, Kelantan, Malaysia; 4 Department of Family Medicine, School of Medical Sciences, Universiti Sains Malaysia, Kelantan, Malaysia; 5 Department of Internal Medicine, School of Medical Sciences, Universiti Sains Malaysia, Kelantan, Malaysia

**Keywords:** module development and validation, education module, insulin adherence, USM-Insulin Adherence Module, USM-IAM

## Abstract

**Background:**

Many patients with type 2 diabetes mellitus (T2DM) do not achieve the desired glycaemic control despite being treated with insulin. Studies found this due to an improper understanding of insulin function, its intensification process and patients’ negative perspective on insulin. We developed an education module to enhance adherence to insulin therapy.

**Methods:**

This study applied a mixed design. It was conducted in three phases: i) Phase I: literature search and focus group discussions (FGDs), ii) Phase II: module development and iii) Phase III: content and face validation of Universiti Sains Malaysia-Insulin Adherence Module (USM-IAM). FGDs were used to gather patients’ opinions. All researchers repeatedly discussed about the module content and arrangement, the words and images used, and the grammar in producing the final draft. Specialists and target audience performed content and face validation of the module.

**Results:**

Thirty-six participants were involved in the FGDs. Data saturation was achieved at the 4th FGD. Three themes emerged from qualitative data analysis and were incorporated into the module. USM-IAM was finalised with five units. The content validity index (CVI) was 0.92, while face validity agreements were between 86% and 97%.

**Conclusion:**

The CVI and face agreement for USM-IAM exceed the cut-off point for a sound module. It has good potential to be used as a resource for educating patients in enhancing insulin adherence.

## Introduction

### Background

Type 2 diabetes mellitus (T2DM) is the most common form of diabetes in adults. Approximately 537 million people globally had diabetes in 2021, a figure that is projected to rise to 783 million by 2045 (1). In addition to a healthy diet and increased physical activity, patients with diabetes mellitus are often prescribed oral glucose-lowering drugs (OGLDs) and/or insulin or injectable agents to achieve glycaemic control. The Malaysia’s local guidelines suggest combining OGLDs with insulin/injectable agents when patients cannot achieve their HbA1c targets after the optimum dosage of OGLDs (2). Despite the development of sodium-glucose cotransporter-2 inhibitors (SGLT2-i) such as canagliflozin and empagliflozin (3) and injectables such as dulaglutide, liraglutide and semaglutide with proven efficacy in reducing HbA1c and cardiac events (4, 5), insulin remains the critical treatment for many patients due to its availability and affordability. Patients with diabetes mellitus will ultimately need insulin 8 years–10 years after the diagnosis of diabetes to maintain a desirable level of glycaemic control (6, 7). Most individuals on a single insulin injection will need intensification within 3 years of insulin initiation (8). Despite the increase in the number of patients using insulin to 65% in a tertiary centre, the percentage of patients who achieve targeted HbA1c levels is still low (9). A literature review revealed that insulin adherence is very poor or unsatisfactory among diabetic patients and is generally lower than the adherence to oral hypoglycaemic agents (10, 11). An internet survey among 502 diabetic patients in America revealed that more than half of them intentionally omitted their insulin and 20% of them omitted insulin regularly (12). A cross-sectional survey of 256 patients with T2DM in Tehran showed that 28.8% of them had low insulin adherence (13). Another cross-sectional study in Japan involving 1,441 patients revealed that 29.4% had low insulin adherence (14). Lastly, a telephone interview survey of 433 subjects in Turkey revealed that 22.03% of patients were non-adherent to their insulin regimes (15).

Many factors contribute to non-adherence to insulin, including pain associated with injections (16), fear of hypoglycaemia and weight gain (17) and fear of embarrassment regarding the administration of insulin in public (18). Reports have emphasised that poor diabetic control results primarily from a lack of understanding of the disease, which leads to the low adherence to proper diabetes management prescribed to patients (e.g. medications or lifestyle modifications) (19), the lack of knowledge on glycaemic targets and the lack of knowledge on diabetes self-care (particularly self-insulin dose adjustment) (20).

Patient education is an integral part of managing diabetes. It has been shown that patients’ education significantly reduces HbA1c (21), blood pressure and cholesterol, and increases diabetes knowledge (22). Patients on insulin require extensive education on the process involved in insulin use and injection; re-education on insulin administration has been shown to improve glycaemic control (23). Patients who had been taught flexible insulin dosing based on their dietary intake and physical activity had better HbA1c reduction levels (24). Thus, in this study, we developed an education module to improve insulin adherence, specifically in patients with T2DM who need insulin. The validation of the module involved the calculation of the content validity index and the patients’ agreement on aspects of the face validity of the module. The relevant insulin therapy guidelines are available in English (25) and are meant for use by healthcare professionals. This newly developed Universiti Sains Malaysia-Insulin Adherence Module (USM-IAM), which uses layperson Malay, could be easily comprehended and used by patients.

## Methods

### Study Design

This study applied a mixed design, employing both qualitative and quantitative methods. It was conducted in three phases: i) Phase I: literature search and focus group discussion; ii) Phase II: module development and iii) Phase III: content and face validation of USM-IAM. Only patients who provided their written informed consent were recruited.

### Phase I: Literature Search and Focus Group Discussion

An extensive literature review was initially conducted to determine whether there were any research papers relevant to this study for module development purposes. A literature search via electronic databases, which includes PubMed, ScienceDirect, EBSCOhost and JSTOR, was carried out for relevant articles on insulin adherence/non-adherence, factors or barriers to insulin adherence and T2DM. Information on barriers to insulin adherence was extracted and summarised. A draft of an education module was constructed based on the information acquired. Patients’ experiences of problems with insulin therapy and suggestions to overcome said problems were sought and gathered through focus group discussions (FGDs) to obtain a complete picture of the situation. A researcher, assisted by a research assistant, screened through the list of patients attending the outpatient clinic and evaluated their eligibility for recruited for the study based on specific inclusion and exclusion criteria. Patients aged 18 years old–75 years old with a formal diagnosis of T2DM by a physician and prescribed insulin for at least 1 year and had HbA1c levels between 8% and 15% were included. Those who could not communicate in Malay, read or write and were not willing/able to give their commitment to this project were excluded.

On the day of the FGD session, patients were given information sheets to read thoroughly, as well as ample time to clarify anything before giving their written consent. Five to seven patients were gathered in a circle prior to the discussion. The investigator explained the rules of discussion. As the investigator asked questions, all patients were expected to answer the questions one after another. They were requested not to interrupt others’ conversations and to respect others’ opinions. Additionally, they were told that there were no right or wrong answers. They were asked not to use their phones during the discussion. All patients consented to the sessions being audiotaped. The questions posed during the FGD followed a natural conversation and were asked in a structured sequence. Most questions were open-ended to encourage patients to provide ideas about their knowledge, problems with insulin therapy and possible solutions.

The interviews were recorded with two digital recorders. A trained assistant took field notes (non-verbal responses such as tone of voice, body language and facial expressions). The sessions continued until a saturation point was achieved (i.e. when patients produced little or no new added information to what had already been discussed). All audiotaped discussions were transcribed verbatim. The transcripts were checked with the original audiotapes to confirm their contents (26). Two researchers (AMZ and NBH) coded the transcripts independently. The gathered data were analysed using a thematic approach (26, 27). NVivo 12 (QSR International Pty. Ltd., Doncaster, Victoria, Australia) was used for data analysis. The list of nodes was grouped into sub-themes and further grouped as themes (Table 2).

### Phase II: Module Development

Module development involved an iterative design and review process accomplished through online meetings with several experts: an endocrinologist (WMIWM), a pharmacologist (AHGR), two family physicians (RM and AMZ), a medical educationist (MZMN) and a pharmacist (NBH). The order of units in the module began with essential diabetic topics and moved on to more insulin-specific topics. Layperson terms were used to communicate effectively with readers. Realistic and culturally appropriate examples, eye-catching images and practical tables were used. During the meetings, these experts discussed the units of the module, the appropriate information to be included, and words to be used in the modules and assessed the suitability of the module’s pictures, charts and tables.

### Phase III: Content and Face Validation of USM-IAM

#### Content Validation by Expert Panels

Based on the published literature, the most extensively used approach for content validity is the content validity index (CVI) (28, 29). We also chose to adopt the CVI for assessment of content validity for the module. We adopted six content validation steps: i) preparing the content validation form, ii) selecting a panel of experts, iii) conducting content validation, iv) reviewing the domain and items, v) scoring each item and vi) calculating the CVI (30).

The content validation form consisted of 20 items, as in the draft module. The review panel was requested to rate the degree of the relevance of each item related to insulin adherence. The score options were placed on a Likert scale from 1 to 4 (1 = not relevant, 2 = somewhat relevant, 3 = quite relevant and 4 = highly relevant) (30). The expert panel was also requested to examine the adequacy of the allocated time duration for each unit. The experts were open to give additional comments for each unit; blank spaces were provided in the form at the end of each unit. It has been recommended that there should be at least five members on the panel to avoid chance agreement during content validation assessment (31). Our panel’s experts were asked to produce ideas from their different specialities (32). The interdisciplinary experts who completed the content validation assessment constituted 10 panellists from different fields of expertise: two endocrinologists, two family medicine specialists, two medical educationists, two pharmacists, a nurse with diabetes training and a dietitian.

These experts were given a hard copy of the content validation form and 2 weeks to complete the task of content assessment. As each item was completed, the scores were transferred to an Excel worksheet for calculation. Items that scored 3 or 4 on the 4-point Likert scale were recorded as 1 (valid) and items that scored 1 or 2 were recorded as 0 (not valid) (33). The number of experts judging an item as relevant (a rating of 3 or 4) was divided by the total number of experts to obtain the CVI, which expresses the proportion of agreement on the relevancy of each item between zero and one (34). It has been proposed that an index of 0.80 or higher is required before an item is accepted or a lower CVI of 0.78 if nine experts are involved (35).

#### Face Validation by Target Audience

To assess the face validity of the USM-IAM, the patients were asked to read the module and complete the face validity form. The patients could ask the investigator questions openly and fill out the form at the allocated time. The instrument aimed at patients consisted of 15 questions focused on the sufficiency of information, the importance and usefulness of information, content legibility, engaging content and helpful illustration. The patients were made to choose either ‘yes’ or ‘no’. The patients were also invited to provide suggestions for module improvement. A total of 20 patients participated in the face validation of the module. The participants included were T2DM patients aged between 18 years old and 75 years old, and prescribed insulin for at least 1 year with HbA1c levels between 8% and 15%. Those who could not communicate in Malay or were illiterate were excluded.

## Results

### Phase I: Literature Search and Focus Group Discussion

#### Socio-Demographic Characteristics

Thirty-six participants involved in the FGDs aged 40 years old–74 years old. The mean age of the participants was 56.7 (7.2) years old; 90% of the participants were Malay and 65% were males; 47.5% had secondary education and 90% were married. Table 1 outlines the demographic characteristics of the participants involved in the FGDs.

#### Emergent Themes

The nodes from the transcriptions were grouped into sub-themes. The sub-themes were further grouped into themes. Data saturation was achieved at the 4th FGD. We continued with additional two FGDs to ensure no more new themes emerged. Three themes that emerged from the FGDs are summarised in Table 2.

We found that patients, generally, have poor knowledge about diabetes.

One patient assumed that an individual has diabetes when their blood sugar level is over 10 mmol/L:

As far as I know, diabetes is when the blood sugar level is more than 10. (P1, FG1 SHC 62-year-old male)

There were also patients with their own classifications of diabetes:

There are two types of diabetes, which are blood diabetes and vein diabetes, (P16, FG3, LLH, a 53-year-old female)

and the third type is nerve diabetes. (P13, FG3, HA, a 60-year-old female)

We noticed that over half of the patients in the discussions had poor knowledge of insulin types and functions. Most patients knew only about the type of insulin they were using. Patients who used premixed insulin knew only one type of insulin, and they were not aware that their insulin contained both prandial and basal insulin and were perplexed when the other members of the group who were on basal-bolus regime were talking about short (clear) and long (cloudy) acting insulins.

As for the other patients injecting insulin, several patients from these group discussions also experienced the adverse effects of insulin. Two-thirds of the patients involved in FGDs had experienced hypoglycaemia. Most of these cases of hypoglycaemia were caused by a reduction in the amount of food ingested, delayed meals after injecting prandial insulin or performing more strenuous physical activities than their usual routine. Most of these patients described having palpitations, sweating, lethargy and hunger, which are symptoms of hypoglycaemia. Other patients described symptoms of confusion (e.g. incorrect prayer recitation) while others had been awakened by their spouses from their sleep, as they had experienced unpleasant dreams and were talking nonsense. Other common adverse effects of insulin experienced by the patients were weight gain, injection site itchiness, pain, bleeding, bruising and generalised allergic reactions.

Several patients on prandial human insulin were bothered at the notion of having to wait 30 min before meals after insulin injections:

The second problem is the time before eating. It is as if we must wait for that time, which means that we must obey the doctor’s instructions. I cannot immediately eat after injecting insulin, and I must be patient. (P7, FG2, KM, a 62-year-old-female)

A patient had incorrect knowledge about proper insulin storage, which made insulin storage troublesome for him:

The problem with injecting insulin is it cannot be taken everywhere. Hot conditions will damage insulin. For example, if I go to Kota Bharu and want to bring insulin, I need to bring it packed in ice, or it will be damaged. (P36, FG6, FLS, a 56-year-old male)

Some patients were not taught about bringing insulin pens when travelling abroad:

It is not easy to bring insulin everywhere. I have been stopped and asked to leave my insulin at the security check in airports. (P32, FG6, MJ, a 59-year-old male)

Some patients forgot to inject:

After coming back from work, I was tired and fell asleep. I recall waking up to inject pre-bed insulin, but I failed to do so. I did not inject until morning (sigh). (P28, FG5, CKS, a 56-year-old female)

Some patients did not taper up their insulin doses and blamed the insulin for being ineffective:

Another problem is that my blood sugar readings were still high after injecting insulin. It occurred despite correct storage, correct technique, and the correct dose prescribed. It makes me think that insulin is not effective. (P13, FG3, HA, a 60-year-old female)

Injecting insulin during fasting will cause hypoglycaemia, generalising it to pre-dawn and pre-iftar doses:

When fasting, I do not inject insulin. I am afraid to get hypoglycaemia. Before sahur, I did not inject insulin, and then I injected the insulin when breaking the fast, but with a reduced dose. (P3, FG1, KM, a 62-year-old female)

### Phase II: Module Development

#### Module Content

The adherence module was developed based on information from the literature and patients’ input from FGDs. As many patients have poor knowledge about diabetes and insulin treatment, the module was drafted to address these problems. Unit 1 provides information on diabetes definition and types, insulin function and types, and its regimes. Unit 2 provides information regarding adherence and the consequences of nonadherence to insulin. The themes from FGD were incorporated into Unit 3: causes of nonadherence and possible solutions; Unit 4: empowering self-care; and Unit 5: fasting safely while undergoing insulin therapy. The dimensions of the USM-IAM draft were 5.8 in × 8.3 in. It contained 36 pages and had a front cover, a back cover, a table of contents and a page for notes. It contained a total of five units. The units, learning outcomes and content of the module are summarised in Table 3.

### Phase III: Content and Face Validation of the USM-IAM

#### Content Validation by an Expert Panel

The panel of experts rated the relevancy of the items. Items that scored 3 or 4 on the 4-point Likert scale were recorded as 1, while and items that scored 1 or 2 were recorded as 0. The CVI of the items is illustrated in Table 4.

The panel suggested classifying hypoglycaemia into mild, moderate and severe and specifying the management of each class. The panel also suggested a few corrections to the vocabulary used. The dietician suggested using pictures for carbohydrate counts for easier understanding. The suggestions were accepted and adopted into the final module. All the raters except for one agreed that the allocated reading time for the module was adequate. The rater stated that patients with lower education levels might require longer than the allocated time to comprehend the module content and suggested that more time be given to them. The experts generally agree that the USM-IAM can be used as a guide for facilitators to conduct education programmes.

The authors reviewed these expert comments. Furthermore, the classification of hypoglycaemia and its management were added. Pictures of carbohydrate exchange are provided in an appendix, and the terminology and grammar were corrected. No new items were added. All 20 items in the second draft of the USM-IAM were maintained under five units.

#### Face Validation by Target Audience

**Figure 1:**
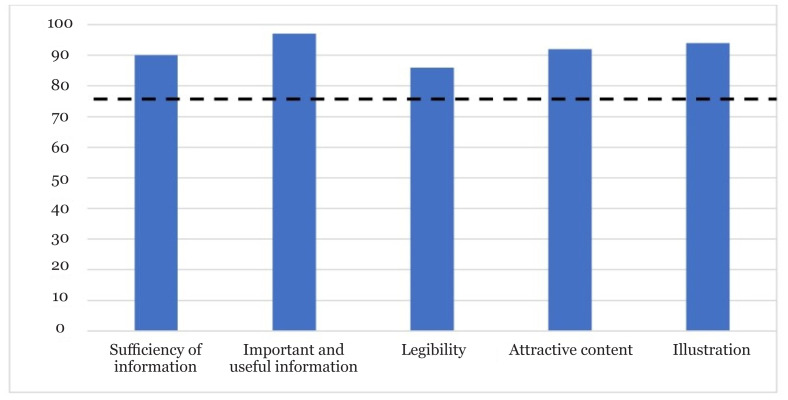
Face validation by target audience

As shown in Figure 1, aspects of face validity achieved a level of agreement between 86% and 97%, which is higher than the required minimum to be valid (75%) (36), indicating an excellent level of agreement among the patients. In addition, the legibility aspect scored lowest (86%) compared to the other aspects.

## Discussion

The present study successfully developed a validated USM-IAM for patients with T2DM. The mean CVI was 0.92, thus confirming content validity. Other studies that validated printed educational modules also utilised the CVI to measure content validity, then underwent revisions until the validated final version was achieved. The process measuring content validity and revising the domain are an important step for the development of quality educational materials (36–38). Modifying educational modules to suit experts’ recommendations is essential to making the module more scientifically accurate and effective for health-learning activities (38).

The USM-IAM content was developed to correct misinformation about insulin among patients. As the cohort of patients interviewed in this study had shown a lack of understanding of the definition of diabetes and its types, as well as the types of insulin available and their varying effects, these were incorporated into the initial part of the module. These were followed by an explanation of the definition of insulin therapy non-adherence and its effects.

Patients with diabetes have many misconceptions about insulin that cause them not to adhere to insulin therapy. For example, the false perception that insulin causes kidney failure, as reported in our FGDs, has also been reported previously (39). Our patient voiced their concern that insulin originated from pork, which is banned for Muslims. This outdated knowledge was corrected by the statement that no more animal-derived insulin exists and that all insulin were produced in labs via bacterial DNA replication.

Patients who experienced adverse effects of insulin adhered less to their insulin regimes compared to those who did not experience adverse effects. As reported in a previous study, our patient cohort also mentioned the side effects of insulin, such as hypoglycaemia, weight gain, injection site problems such as pain, bleeding, bruising, injection site scarring and allergic reactions (12, 15, 39). Insulin injection is bothersome for some patients, particularly those on human insulin. Some had problems with damaged insulin due to improper transportation and storage. A few of them were stopped from bringing their insulin on board aeroplanes, not to mention those who forgot to take their insulin shots, used incorrect injection techniques or were reluctant to inject insulin in public to avoid exposing the ‘aurat’ (body parts that Muslims are forbidden from exposing to others).

It is vital to address all these difficulties to improve adherence to insulin. These issues were delivered in the third module unit: causes of insulin nonadherence and measures to overcome them. Many patients did not achieve glycaemic control despite insulin usage, as they lacked the knowledge and understanding of their treatment purpose, targets and the need for and techniques of insulin intensification (19, 40, 41). These issues were addressed in the fourth module unit on empowering self-care. Last but not least, an important subject covered in the module concerns fasting safely. It is essential to cover this topic, as most of our patients are Muslims, and they will fast even if the doctors classify them as being at moderate or high risk and advise them against fasting (42). These patients should be aware of the fact that complications among high-risk patients are higher during Ramadan than at other times of the year (43).

The strength of the USM-IAM is that it was tailored to the needs of Malaysian T2DM patients treated with insulin therapy. The USM-IAM was field-tested among potential patients and had its content validated by an expert panel before being implemented in an intervention study. This enhanced the effectiveness of the module as content refinement was accomplished.

Overall, the CVI of the USM-IAM was 0.92, which was considered good—it was higher than the published physical activity educational material’s CVI of 0.85 (37) but was lower than the booklet for childbirth companions, which had a CVI of 0.94 (36). It has been proposed that an index of 0.80 or higher is required before an item is accepted (a lower CVI of 0.78 if required nine experts are involved) (35). The USM-IAM achieved an agreement rate ranging from 86% to 97% for face validity compared to the physical activity education module (75.6% to 86.7%) and childbirth companion module (81.8% to 100%). The acceptable agreement rate for face validity is >75% (36). The practice of validating education modules using CVI and face validity index has been widely accepted as a tool to validate printed education modules (29). It is a practical approach—the content derived from the module was incorporated into the form and judged by experts. Content judged by experts to be unrelated should be removed before the development of the final module (34).

With the development of many educational models and statistical applications, newer researchers have adopted these models and applications in the development and validation of their modules. Educational material developed in a nurse-led self-management education in adults with T2DM used the Taba model (44), which is one of the few models for curriculum development. Seven basic steps of Taba models are diagnosis of learners’ needs, formulation of objectives, selection of the content., organisation of the content, selection of learning activity, organisation of learning activities and evaluation. These models are too complex (45), and the steps proposed in these models do not apply to this study as the authors had decided the learning activity is through counselling given by diabetes educators. The educational material for diabetes self-management education among patients with T2DM in Sri Lanka was validated in terms of judgment and criteria validity, including sensitivity, specificity and the area under the receiver operating characteristic (ROC) curve (46). Testing the sensitivity and specificity of a new tool for medical diagnosis is crucial. However, the need to determine the sensitivity and specificity of educational content is arguable. An integrated diabetes-periodontitis education module that contains 17 infographic flip charts and 13 short videos was validated using PEMAT (47), which is suitable for validating multimedia content under its subsection PEMAT-A/V (48), compared to CVI which did not specifically assess multimedia content.

## Conclusion

The USM-IAM was explicitly developed to enhance patients’ adherence to insulin. It has high content validity and face validity, reflecting its impactful content and patient acceptance. It has the potential to be used as a resource for educating patients in enhancing their insulin adherence.

## Figures and Tables

**Table 1:** Sociodemographic profiles of the 36 patients involved in FGD

Characteristics	FG1	FG2	FG3	FG4	FG5	FG6	*n*	%
Gender								
Male	4	4	4	2	3	5	22	61.1
Female	1	3	3	4	3	0	14	38.9
Ethnicity								
Malay	5	7	5	6	5	4	32	88.9
Others	0	0	2	0	1	1	4	11.1
Marital status								
Single	0	0	0	1	0	0	1	2.8
Married	5	7	6	3	6	5	32	88.9
Widowed	0	0	1	2	0	0	3	8.3
Occupation								
Pensioner	3	1	0	2	2	4	12	33.3
Government servant	1	4	4	0	1	0	10	27.8
Self-employed	0	0	2	1	0	1	4	11.1
Housewife	1	2	1	3	3	0	10	27.8
Household monthly income (RM)								
< 3,000	3	4	5	6	6	4	28	77.8
3,001–6,000	2	1	0	0	0	0	3	8.3
6,001–13,000	0	2	2	0	0	0	4	11.1
> 13,000	0	0	0	0	0	1	1	2.8
Duration of diabetes (years)								
< 5	0	1	1	1	0	1	4	11.1
5–9	0	1	1	1	1	0	4	11.1
10–15	1	1	1	3	2	1	9	25.0
> 15	4	4	4	1	3	3	19	52.8
Number of injections/days								
One	0	1	3	0	0	1	5	13.9
Two	2	3	0	0	2	1	8	22.2
Three	0	2	2	1	1	2	8	22.2
Four	3	1	2	5	3	1	15	41.7

**Table 2:** Themes emerged from FGD

Theme	Sub-themes	Nodes
Poor knowledge	Poor knowledge	Poor knowledge on definition and types of diabetes
		Misunderstanding about the function of insulin
		Poor knowledge on insulin types and their respective actions

Problems with insulin therapy	Side effect of insulin	Hypoglycaemia
		Weight gain
		Allergic reaction

	Injection site problems	Pain/scarring/fibrosis
		Bleeding/bruising

	Attitude towards insulin	Insulin restricts daily activity
		Forgetting to inject

		Embarrassed to inject in public

	Financial constraint	High cost of glucose strips and needles

	Misperception of insulin	Insulin is ineffective
		should not inject when fasting
		Forbidden to carry insulin when travelling on the aeroplane

	Myths	Insulin is derived from porcine sources. Forbidden for Muslim
		Insulin causes kidney failure

Possible solutions	Taking meals within 20 min of injecting insulin	Has hypoglycaemia after injecting pre-lunch insulin. This can disturb tasks

	Refrain from consuming excessive food to avoid weight gain	Binge eating as always feel hungry
		Excessive weight gain

	Rotate the injection sites to avoid scarring	Scarring/hardened area
		Fibrosis

	To find a confined space to inject insulin	Embarrassed to inject in public

	Take specific measures to overcome forgetfulness	Forgetting to inject insulin
		Forget to bring insulin pen to work

**Table 3:** Topics, learning outcomes and content of USM-IAM

Unit	Topics	Learning outcomes	Content
1	Diabetes and insulin	To give general information to diabetic patients about diabetes and its relationship with insulin	Diabetes definition Types of diabetes The relationship between diabetes and insulin Types of insulin Why diabetics inject different insulin types Insulin regimes
2	Non-adherence to insulin treatment and the consequences of non-adherence	Improve the knowledge and understanding of diabetic patients about non-adherence to insulin treatment and its outcome	Definition of nonadherence to insulin treatment The outcome of non-adherence to insulin treatment
3	Causes of non-adherence to insulin treatment and suggested solutions	Improve the knowledge and understanding of diabetic patients about the causes of non-adherence to insulin injection and how to overcome them	Insulin side effects Problems with insulin injections Negative attitude towards insulin Expensive cost for monitoring Wrong perception on insulin Myths on insulin
4	Empowering diabetes self-care	Improve knowledge and understanding of target sugar control, blood pressure, cholesterol, and ideal body weight. Improve the motivation of diabetic patients to monitor sugar levels and adjust insulin doses to changes in blood glucose levels	Be disciplined in controlling diabetes Do self-monitoring of sugar levels Changing the insulin dose based on their sugar level
5	Fasting safely with insulin treatment	Improve the knowledge of diabetic patients about methods to fast safely despite injecting insulin	When should I monitor my sugar levels while fasting? How to modify the insulin dose while fasting? When should I break my fast?

**Table 4:** Items relevancy judged by expert panels

		Rater 1	Rater 2	Rater 3	Rater 4	Rater 5	Rater 6	Rater 7	Rater 8	Rater 9	Rater 10		Experts in agreement	I-CVI	UA
	Item														
1	What is diabetes?	1	1	1	1	1	0	1	0	1	1		8	0.8	1
2	How many types of diabetes?	1	1	1	1	1	1	1	0	1	1		9	0.9	0
3	What is the relationship between diabetes and insulin	1	0	1	1	1	1	1	0	1	1		8	0.8	0
4	Why patients inject different insulin types?	1	1	1	1	1	1	1	0	1	1		9	0.9	0
5	How many types of insulin?	1	1	1	1	1	1	1	0	1	1		9	0.9	0
6	Insulin regimes	1	1	1	1	1	1	1	0	1	1		9	0.9	0
7	Definition of insulin non-adherence	1	1	1	1	1	1	1	1	0	1		9	0.9	0
8	Consequence of insulin non-adherence	1	1	1	1	1	1	1	1	1	1		10	1	1
9	Insulin side effects	1	1	1	1	1	0	1	1	1	1		9	0.9	0
10	Problems with insulin injections	1	1	1	1	0	1	1	1	1	1		9	0.9	0
11	Negative perception toward insulin	1	1	1	1	1	1	1	1	1	1		10	1	1
12	High cost	1	1	1	1	1	1	1	1	1	1		10	1	1
13	Wrong perception toward insulin	1	1	1	1	1	1	1	1	1	1		10	1	1
14	Myths on insulin	1	1	1	1	1	1	1	1	1	1		10	1	1
15	Be disciplined in controlling diabetes	1	1	1	1	1	1	1	1	1	1		10	1	1
16	Do self-monitoring of sugar levels	1	1	1	1	1	1	1	0	1	1		9	0.9	0
17	Change the insulin dose based on the sugar level	1	0	1	1	1	1	1	0	1	1		8	0.8	0
18	When should I monitor my sugar levels while fasting?	1	1	1	1	1	1	1	1	1	1		10	1	1
19	How to modify the insulin dose while fasting?	1	1	1	1	1	1	1	1	1	1		10	1	1
20	When should I break my fast?	1	1	1	1	1	1	1	1	1	1		10	1	1
													S-CVI/Ave	0.923	
	Proportion relevant	1.00	0.90	1.00	1.00	0.95	0.90	1.00	0.57	0.95	1.00		S-CVI/UA		0.48
				
				Average proportion of items judged relevant across the 10 experts								0.92			

Notes: Items scored as 3 or 4 on 4-Point Relevance Scale recorded as 1, item scored as 1–2 recorded as 0
